# Heterologous expression of a tannic acid-inducible laccase3 of *Cryphonectria parasitica *in *Saccharomyces cerevisiae*

**DOI:** 10.1186/1472-6750-10-18

**Published:** 2010-02-24

**Authors:** Jung-Mi Kim, Seung-Moon Park, Dae-Hyuk Kim

**Affiliations:** 1 Institute for Molecular Biology and Genetics, Center for Fungal Pathogenesis, Chonbuk National University, Jeonju, Chonbuk 561-756, Korea

## Abstract

**Background:**

A tannic acid-inducible and mycoviral-regulated laccase3 (*lac*3) from the chestnut blight fungus *Cryphonectria parasitica *has recently been identified, but further characterization was hampered because of the precipitation of protein products by tannic acid supplementation. The present study investigated the heterologous expression of the functional laccase3 using a yeast *Saccharomyces cerevisiae*.

**Results:**

Laccase activity in the culture broth of transformants measured using a laccase-specific substrate suggested that the *lac*3 gene was successfully expressed and the corresponding protein product secreted into the culture media. In addition, activity staining and Western blot analysis of a native gel revealed that the enzyme activity co-existed with the protein product specific to anti-laccase3 antibody, confirming that the cloned *lac*3 gene is responsible for the laccase activity. When transformants were grown on plates containing tannic acid-supplemented media, brown coloration was observed around transformed cells, indicating the oxidation of tannic acid. However, the enzymatic activity was measurable only in the selective ura^- ^media and was negligible in nonselective nutrient-rich culture conditions. This was in part because of the increased plasmid instability in the nonselective media. Moreover, the protein product of *lac*3 appears to be sensitive to the cultured nonselective nutrient-rich broth, because a rapid decline in enzymatic activity was observed when the cultured broth of ura^- ^media was mixed with that of nonselective nutrient-rich broth. In addition, constitutive expression of the *lac*3 gene resulted in a reduced cell number of the *lac*3 transformants compared to that of vector-only transformed control. However, the presence of recombinant vector without *lac*3 induction did not affect the growth of transformants.

**Conclusions:**

The results suggest that expression of the *lac*3 gene has an inhibitory effect on the growth of transformed *S. cerevisiae *and that the controlled expression of *lac*3 is appropriate for the possible application of recombinant yeast to the treatment of phenolic compounds.

## Background

Laccases are multi-copper-binding phenoloxidases (EC 1.10.3.2) that were first detected in the Japanese lac tree *Toxicodendron verniciflua*; they are also found in certain other plants as well as many insects and a variety of fungi [1-3]. Laccases are particularly widespread in ligninolytic basidiomycetes, and more than 125 different basidiomeceteous laccase genes have been described [4]. The biological functions of laccase in fungi are diverse, as laccase is implicated in various cellular processes, including delignification [5,6], sporulation [7], pigment production [8-10], fruiting body formation [7], and pathogenesis [11,12].

Using oxygen as the final electron acceptor, laccases catalyze the oxidation of a number of aromatic substances such as diphenols, methoxy-substituted monophenols, and aromatic amines [13]. Many laccases are characterized by the presence of one type-1, one type-2, and two type-3 copper ions. One electron at a time is removed from the substrate by a type-1 copper ion and is transferred to the type-2/type-3 copper site, where molecular oxygen is reduced to water [14]. Because of their low substrate specificity, industrial applications for laccases include delignification [15], the purification of colored waste water [16], textile dye decoloration [17], beverage and food treatment [18], the sulfurization and solublization of coal to their use in enzyme-based biosensors [19], and the transformation and inactivation of toxic environmental pollutants [20]. In addition, recent studies have shown that the substrate specificity of the enzyme can be broadened in the presence of redox mediators [21]. Given the versatility and broad spectrum of substrate specificity, laccases could become among the most important biocatalysts in fungal biotechnology [3].

At least three different laccases are present in chestnut blight fungus *C. parasitica *[22]. Among these, laccase3 is of interest because it is induced specifically by the presence of tannic acid but not by other commonly known fungal laccase inducers such as ferulic acid and 2,5-xylidine, which are structurally related to lignin [11]. In addition, given that *C. parasitica *is a necrotic fungus rather than a wood-decaying fungus, laccase3 is unique to *C. parasitica *and is predicted to be involved in overcoming tannic acids, an abundant group of phenolic compounds found in the bark of chestnut tree that function as a natural barriers against pathogen infection. The phenolic metabolism in plants is complex and yields a wide array of compounds ranging from flower pigments to the complex phenolics of the plant cell wall lignin. However, the group of phenolics known as tannic acids is clearly distinguished from other plant phenolics in terms of chemical reactivity and biological activity [23]. Tannic acids are water-soluble phenolic compounds with molecular weights between 500 and 3,000 that exhibit distinct properties such as the ability to precipitate alkaloids, gelatin, and other proteins [24]. The characteristic that sets tannic acids apart from all other phenolics is their ability to precipitate proteins. Therefore, having an enzyme capable of degrading tannic acid or that is insensitive to the presence of tannic acid may be useful for various applications of plant tissue materials. Thus, producing laccase3 on an industrial scale may be useful for processing plant materials with high contents of tannic acid.

Because laccases are notoriously difficult to express in nonfungal systems [25], several fungi, including *Saccharomyces cerevisiae *[25,26], *Trichoderma reesei *[27], *Aspergillus oryzae *[28], *Pichia pastoris *[29], *Kluyveromyces lactis *[26], *A. sojae *[30], and *A. niger *[31], were used for the heterologous expression of laccase. Although *P. pastoris *has been used as a heterologous host for laccase more often than *S. cerevisiae *in yeast because of its higher production levels, successful expression in *S. cerevisiae *has been reported [32]. In addition, *S. cerevisiae *has a long history of application as a GRAS (Generally Recognized As Safe) organism without the integration of expression constructs. Therefore, we report here the heterologous expression of a novel laccase3 using *S. cerevisiae *and its effects on the transformed *S. cerevisiae*.

## Results

### Analysis of transformed *S. cerevisiae*

From 10 to 20 transformants of *S. cerevisiae *representing each recombinant plasmid were selected on ura^- ^medium. Colony PCR using the DNA preparations from these putative transformations revealed the presence of amplified 1.8-kb DNA fragment, indicating that the recombinant plasmid was transferred into yeast. In addition, transformation of *E. coli *using DNA preparations from all selected transformants confirmed the presence of the recombinant plasmid in yeast.

### Expression of LAC3 protein using constitutive promoter

**Figure 1:**
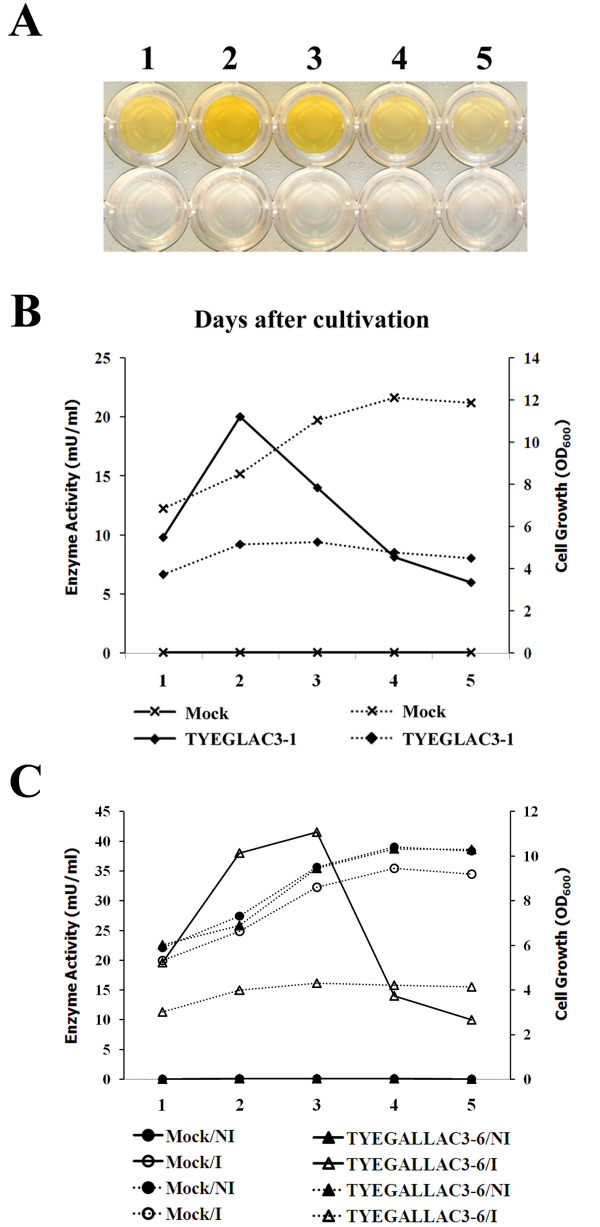
**Expression of laccase3 from recombinant *S. cerevisiae *TYEGLAC3-1 and TYEGALLAC3-6**. (**A**) Brown coloration of DMOP substrate by the recombinant laccase3 in the culture supernatant of TYEGLAC3-1. Colormetric detection after 1 h in a reaction mixture is shown. Numbers above each well indicate the number of days after cultivation. Results of vector-only transformants are shown at corresponding lower lines of wells as a control. Cell growth (dotted line) and laccase3 activity (solid line) in the culture filtrate of recombinant *S. cerevisiae *TYEGLAC3-1 (**B**) and TYEGALLAC3-6 (**C**) at various days after cultivation. YEPD (non-induction) and galactose-supplemented glucose-free ura^- ^(induction) media are indicated after the strain designation as NI and I, respectively. Cell growth was monitored by measuring OD_600 _with a spectrophotometer. Vector-only transformed (Mock) cells were included as a control.

**Figure 2:**
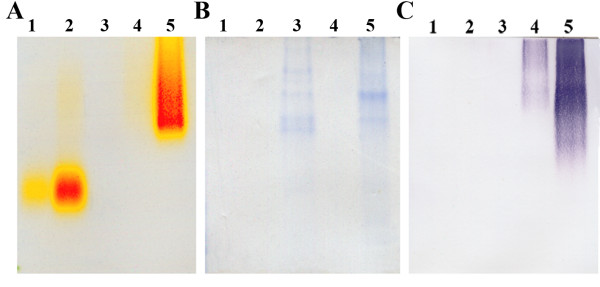
**Western blot analysis and native PAGE activity staining**. Culture supernatant 2 days after cultivation was resolved on native PAGE followed by activity staining using the substrate solution (**A**). Coomassie staining (**B**) and Western blot analysis of the twin gel (**C**) were performed. Lanes 1 and 2 contain 1 and 5 mU laccase, respectively, from *Trametes versicolor *as controls. Lane 3 contains 12 μl 40× concentrated 3-day-old culture supernatant of a mock transformant. Lanes 4 and 5 contain 12 μl of non-concentrated and 40× concentrated, respectively, 3-day-old culture supernatant of TYEGLAC3-1.

Expression of the transgene encoding laccase3 directed by the *GPD *promoter was examined by enzymatic assay using culture supernatant and DMOP as enzyme and substrate, respectively. When culture supernatants of transformants grown in YEPD media for 3 days were measured for enzymatic activity, no laccase activity was observed. However, when culture supernatants were prepared from the ura^- ^selective broth, all transformants showed laccase activity. There were variations in laccase activity among the strains transformed with the same expression construct. A wide variation in heterologous gene expression in *S. cerevisiae *is not unusual when episomal 2 μ ori-based plasmids are used, possibly because of variation in the plasmid copy number between different transformants [33,34]. Thus, transformant TYEGLAC3-1 showing the highest enzyme activity was selected for further analysis. Transformant TYEGLAC3-1 was cultured for 5 days in the ura^- ^selective media, and samples were analyzed to obtain the temporal expression pattern of recombinant laccase3 (Figure 1A). The highest laccase activity was 20.0 mU/ml culture supernatant detected after 2 days of cultivation. Thereafter, a rapid decrease in enzyme activity was detected as the culture progressed (Figure 1B). Native gel electrophoresis of culture supernatant followed by staining with DMOP substrate solution revealed the presence of a band showing the laccase activity (Figure 2A). Coomassie blue-staining of the native gel revealed the presence of a protein band at the corresponding position (Figure 2B). In addition, Western blot analysis showed the immuno-reactivity of the corresponding protein band (Figure 2C). Again, neither laccase activity nor an immuno-specific band was observed in the culture supernatant of transformant TYEGLAC3-1 grown in nonselective media and nutrient-rich YEPD media. These results indicate that ura^- ^selection is required for the transformant to show laccase activity, which is maintained in ura^- ^selective cultured media. Moreover, they also indicate that laccase activity in the culture supernatant is due to the protein product of the *lac*3 gene (Figure 2).

**Figure 3:**
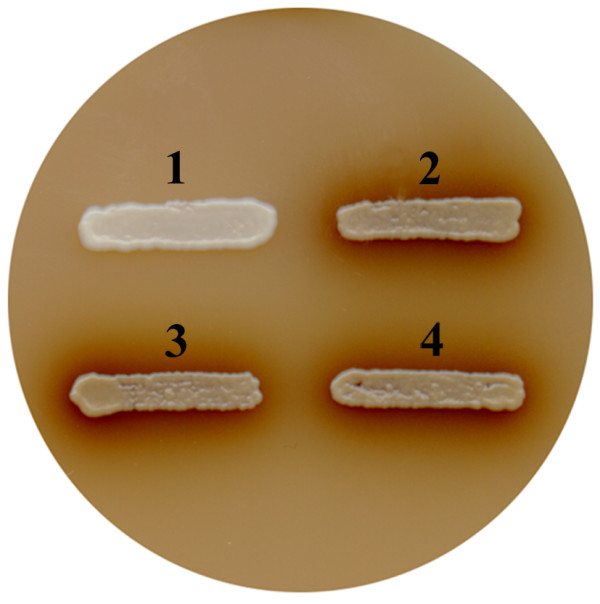
**Plate assay using tannic acid-supplemented media**. Dark brown staining was observed around colonies of transformants, whereas no color reaction was observed around a colony of a mock transformant. 1, 2, 3, and 4 represent the mock transformant, TYEGLAC3-1, TYEGLAC3-5, and TYEGLAC3-7, respectively.

Because the laccase3 from *C. parasitica *is responsive to tannic acid, oxidation of tannic acid by the expressed laccase3 was measured using a tannic acid-supplemented medium. Plates containing 0.5% tannic acid-supplemented ura^- ^selective medium were prepared, and transformed cells were grown. As shown in Figure 3, transformants showed dark brown coloring around the colonies, whereas no color reactions were observed around the vector-only transformed cells (mock transformants). This indicates that transformants are able to express and secrete laccase3 into medium and that the expressed recombinant laccase3 maintains biochemical characteristics of oxidizing a phenolic compound such as tannic acid.

### Plasmid stability and cell growth of recombinant strains

Plasmid stability and cell growth were assessed by measuring CFUs on nonselective YEPD as well as selective ura^- ^media and by counting the number of cells using Haemacytometer, respectively. The transformant TYEGLAC3-1 and two other randomly selected transformants, TYEGLAC3-5 and -7 were examined for plasmid stability. When the plasmid stability of transformants grown in YEPD broth was measured by plating 200 ul 10^6 ^diluted 3-day-old culture suspension on a ura^- ^selective plate and a nonselective YEPD plate, more than 65% of cells appeared to lose plasmid after the 3-day incubation (Table 1). This number was significantly higher than that of mock transformants and of previous studies using similar plasmids [34,35]. Although it was not as high as for YEPD media, this high plasmid instability was also observed in the cells grown in ura^- ^selective medium, in which 50% of plated cells were not able to grow to form distinct colonies.

**Table 1:** CFUs of recombinant strains on YEPD and ura^- ^selective media.

Strain	CFUs/plate^**1**^	
	
	YEPD^**2**^	ura^**-**^
Mock transformant		
YEPD plating	917 ± 27 ^3^	892 ± 22
ura^- ^plating	819 ± 16	813 ± 24
TYEGLAC3-1		
YEPD plating	302 ± 17	189 ± 6
ura^- ^plating	98 ± 5	100 ± 4
TYEGLAC3-5		
YEPD plating	311 ± 14	217 ± 8
ura^- ^plating	103 ± 3	112 ± 4
TYEGLAC3-7		
YEPD plating	351 ± 11	218 ± 7
ura^- ^plating	112 ± 5	112 ± 3

^1 ^Colony-forming units per plate

^2 ^For each strain, 1 × 10^7 ^cells were inoculated into corresponding media, as described in Materials and methods.

^3 ^Five replicates of each strain were used, and each experiment was repeated three times. Values are shown as the means ± standard deviation.

Cell growth was also compared between *lac*3 transformants and a mock transformant. Compared to the mock transformant, which exhibited 917 CFUs on a YEPD plate, *lac*3 transformants exhibited less than 40% growth. In addition, when cells were grown in ura^- ^selective broth, further reduction in cell growth (≥ 25% of CFUs) was observed compared to that of a mock transformant (892 CFUs; Table 1).

### Effect of cultured broth on expressed LAC3

Even though the number of cells harboring the plasmid was significantly reduced during cultivation of the TYEGLAC3-1 transformant in nonselective YEPD media, a large number of cells still contained the plasmid. It was interesting that no laccase activity was detected in the cultured supernatant of YEPD. We therefore examined enzyme stability by measuring enzyme activity followed by mixing the supernatant of ura^- ^selective broth containing laccase activity with the supernatant of cultured YEPD broth. After 24 h of incubation at 25°C, no laccase activity was observed. However, mixtures with fresh YEPD media or reaction buffer showed laccase activity, indicating that expressed laccase activity is sensitive to cultured YEPD media, whereas laccase activity in the ura^- ^selective media is stably maintained.

**Figure 4:**
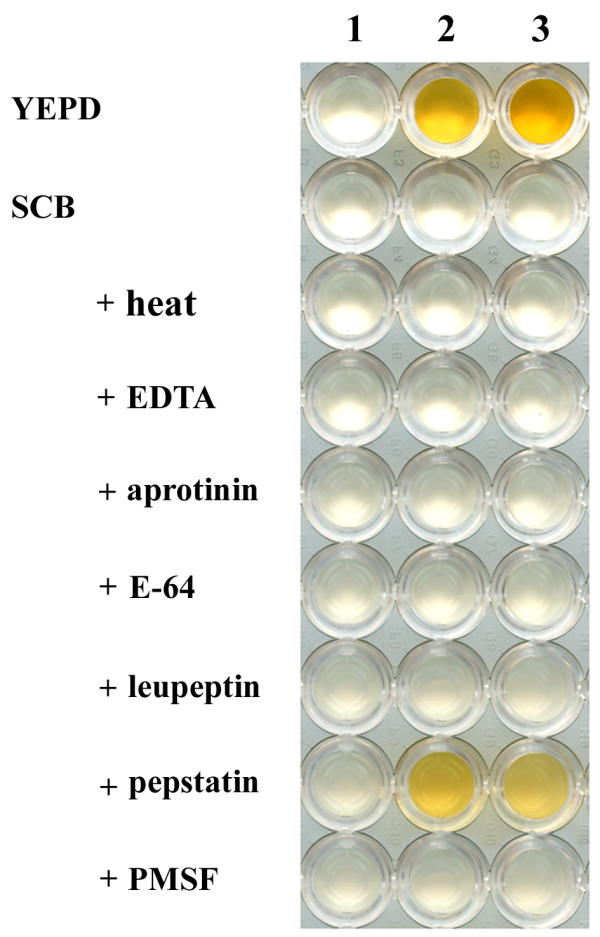
**Protection of laccase3 activity by various protease inhibitors**. Brown coloration of DMOP substrate by preincubated ura^- ^selective media and colormetric observation was as described in Figure 1. Lanes 1-3 contain the 3-day-old ura^- ^culture supernatant of a mock transformant, TYEGLAC3-1, and TYEGLAC3-5, respectively. YEPD and SCB represent the additions of fresh YEPD media and supernatant of TYEGLAC3-1-cultured YEPD broth, respectively. + indicates various protease-inhibiting treatments to SCB. Six different protease inhibitors [aprotinin (0.8 μM), E-64 (84.0 μM), EDTA (0.8 mM), leupeptin (10.0 μM), pepstatin (7.3 μM), and PMSF (2.0 mM)] were tested and incubation at 65°C for 30 min was conducted as heat treatment [41]. Note that the addition of pepstatin maintains the browning, whereas no other protease inhibitors do.

The instability of expressed laccase activity in the cultured YEPD media was examined using various protease inhibitors. As shown in Figure 4, laccase activity in the supernatant of ura^- ^selective broth was abolished when the medium was mixed with supernatant of cultured YEPD broth. However, when the cultured YEPD medium was preincubated with pepstatin, an aspartyl protease inhibitor, and then mixed with the supernatant of ura^- ^selective broth containing laccase activity, residual laccase activity was observed, although it was not as distinct as that of the control. No protection was observed aprotinin, EDTA, E-64 protease inhibitor, leupeptin, or PMSF.

### Expression of LAC3 protein using an inducible GAL1 promoter

Expression of the transgene encoding laccase3 directed by an inducible *GAL*1 promoter was examined. When transformants harboring the plasmid pYEGALLAC3 were cultured for 5 days in 2% galactose-containing inducing media, culture supernatants of all transformants showed the enzyme activity (Figure 1C). A primary inoculum was prepared from 5 ml appropriate media to maintain selection but with 2% raffinose/0.1% glucose as the carbon source. There were variations in laccase activity among the strains transformed with the *GAL*1 promoter expression construct as well. The transformant TYEGALLAC3-6, which showed the highest enzyme activity at 41.5 mU/ml culture supernatant, was selected for further study. Compared to the transformant TYEGLAC3-1 expressing the *lac*3 gene by the constitutive *GPD *promoter, transformants using an inducible *GAL*1 promoter revealed higher levels of laccase activity. In addition, no growth defects of the transformant TYEGALLAC3-6 in YEPD media were observed (Table 2), suggesting that no growth defect was observed unless laccase expression occurred. The plasmid stability of the introduction of pYEGALLAC3 to the selected transformant TYEGALLAC3-6 was good, as more than 85% of the cells retained the plasmid after the 3-day incubation in nonselective media. These results suggested that the stability of the plasmid was maintained unless laccase expression was induced.

**Table 2:** CFUs of recombinant strains TYEGALLAC3-6 on induction and non-induction media.

Strain	CFUs/plate^**1**^	
	
	YEPD^**2**^	ura^**-**^/Gal
Mock transformant		
YEPD plating	792 ± 28 ^3^	749 ± 11
ura^- ^plating	763 ± 13	711 ± 23
TYEGALLAC3-6		
YEPD plating	768 ± 15	391 ± 21
ura^- ^plating	690 ± 17	175 ± 15

^1 ^Colony-forming units per plate

^2 ^For each strain, 1 × 10^7 ^cells were inoculated into corresponding media, as described in Materials and methods. YEPD and galactose-supplemented glucose free ura^- ^selective media (ura^-^/Gal) were used as non-induction and induction media, respectively.

^3 ^Five replicates of each strain were used, and each experiment was repeated three times. Values are shown as the means ± standard deviation.

## Discussion

Laccase catalyzes one-electron oxidation of diverse substrates with the concomitant reduction of molecular oxygen to water by the transfer of four electrons. Laccases are divided into "low-redox potential" and "high-redox potential" laccases depending on the structure and properties of the copper centers [3]. We recently characterized a new laccase, laccase3, which is induced by the presence of tannic acid [11]. Sequence comparison indicates that laccase3 belongs to the "low-redox potential" group, which is more common in fungi with the exception of white-rot fungi [36]. Given that this enzyme is specific to tannic acid and represents a new laccase from necrotic but not wood-decaying fungus, characterizing its biochemical properties is of interest. However, like most other laccases produced by fungi, its expression level is far too low, and the presence of tannic acid appears to be a limiting factor for protein purification because it precipitates the protein. Therefore, the heterologous expression of functional laccase3 is required for a better understanding and application of this enzyme.

Compared to previous studies that used *S. cerevisiae *as a laccase expression host, the present study showed a relatively high maximum enzyme activity of 41.5 U/L [25,37,38]. However, improved laccase production using *S. cerevisiae *was achieved by direct evolution and expression of the mature form of laccase, which replaced the endogenous prepropeptide with that of the *S. cerevisiae *α-factor and removed the inhibitory C-terminal extension [25,32]. Laccase3 from *C. parasitica*, like some other laccases from ascomycetes, does not have a C-terminal extension and exhibits C-terminal ends with the last four amino acids of Asp-Ser-Gly-Ile, which represent the processing site [32]. Thus, replacing the endogenous laccase signal peptide with the rice amylase1A signal peptide represents the engineered portion of the recombinant laccase3 gene construct in this study, which may be further manipulated to produce economical levels of expression. In addition to conventional laccase activity on a commonly known substrate such as DMOP, yeast-expressed laccase3 showed the characteristic enzyme activity on tannic acid, which was visualized by coloring the tannic acid-supplemented media on which the recombinant strains expressing laccase3 were grown. Therefore, these results potentiate the possible application of the recombinant yeast strain for treating phenolic waste even in the presence of tannic acid. In addition, the plate assay based on the color reaction showed a clear contrast; i.e., recipient cells showed no browning around the colonies, but dark browning around the colonies was observed in transformants. As in the case of the Bavendamm assay, used to measure the phenol oxidase activity, the area of the colored zone appears to correlate with enzyme activity [39]. Therefore, this test can be further applied as a simple screening method for selecting transformants with higher laccase activity.

Selection pressure to maintain the recombinant vector designed to express the laccase constitutively resulted in a growth defect as detected by the reduced number of cells. However, harboring the vector only without the laccase gene or maintaining the recombinant vector designed to induce the laccase expression under non-inducing conditions did not result in any growth defects. In addition, no significant growth defect was observed by expressing other foreign protein products using a similar constitutive promoter [34,35]. Moreover, growth defects of transformants using an inducible promoter for laccase expression were observed only when transformants were cultured under induction conditions. Therefore, expression of recombinant laccase3 appears to be harmful to the host cell. This is in accordance with most previous studies, in that an inducible instead of a constitutive promoter was used for the successful expression of laccase in yeast. Like other ligninolytic enzymes, laccases are notoriously bad at producing large amounts as recombinant proteins [40]. One of the possible limitations to the mass production of recombinant laccase is the harmful effect on cell growth observed in this study. Therefore, controlled expression of laccase via the application of an inducible promoter is suggested for the large-scale production of recombinant laccase. Further studies examining the effect of laccase expression on the growth of *S. cerevisiae *will be of interest.

The expressed protein product of laccase3 was labile to the cultured media of nutrient-rich YEPD. The instability of expressed laccase activity could be due in part to the protease activity in cultured media, because pepstatin (for aspartyl protease) was able to preserve some residual laccase activity. The lack of protection using other protease inhibitors such as aprotinin (for serine protease), EDTA (for metallo protease), E-64 (for cystein protease), leupeptin (for serine/cystein protease), and PMSF (for serine protease) suggests that the residual protease activity is due to the presence of an aspartyl protease, which is expressed under some conditions of fermentation [41]. It is of interest to see that the host strain *S. cerevisiae *2805 is a mutant at the *pep4 *gene encoding protease A (an aspartyl protease). Strain lacking protease A accumulate inactive precursors of protease B and carboxypeptidase Y and show reduced activity of a number of other hydrolases, which consequently results in a significant reduction of overall proteolysis [42,43]. Therefore, another aspartyl protease may contribute in part to the instability of the laccase. However, there may to be additional reasons for the instability of laccase beyond the protection of residual laccase activity by pepstatin. Whether because of protease activity or other unknown reasons, the instability of laccase seems to be specific rather than general, because the same host strain is used for the expression of various proteins, including enzymes, cytokines, and structural proteins [33,35,44], without noticeable instability of expressed target proteins. Therefore, along with the controlled expression of laccase in the recombinant strain, culture conditions that minimize the degradation of expressed laccase, such as using defined simple media rather than complex media, could be applied to improve the production of recombinant laccase3 and to further apply recombinant strains to treat phenolic compounds.

## Conclusions

Taken together, this study demonstrated the heterolgous expression of a novel tannic acid-inducible laccase3 and described why laccase expression on commercial scale is difficult. In addition, we have suggested a possible method of producing a large amount of recombinant laccase and discussed the potential of the recombinant strain for bioremediation.

## Methods

### Strains and culture conditions

Plasmids were maintained and propagated in *Escherichia coli *HB101 or DH5α as described in Sambrook *et al*. [45]. The cDNA encoding laccase3 from *Cryphonectria parasitica *was obtained from our previous study [11], and *S. cerevisiae *2805 (*MATα pep4::HIS3 prb1-δ can1 GAL2 his3-200 ura3-52*) was used for heterologous production of *lac*3 [44].

*S. cerevisiae *was maintained in YEPD medium (yeast extract, 10 g/l; peptone, 20 g/l; dextrose, 20 g/l). Uracil-deficient (ura^-^) selective medium (6.7 g/l yeast nitrogen base without amino acids, 0.03 g/l adenine and tryptophan, 5 g/l casamino acid, 20 g/l dextrose, 20 g/l agar) was used to screen transformants at 30°C. A primary inoculum was prepared from 5 ml uracil selective medium cultured for 24 h, and 1 × 10^7 ^cells were transferred to a 300-ml Erlenmeyer flask containing 40 ml of either YEPD broth or ura^- ^selective broth. Expression cultures were grown at 30°C with continuous agitation (200 rpm) for 2 - 3 days, after which culture supernatants were obtained by centrifugation at 3,000 *g *and were assayed for laccase activity as described previously [22].

### Plasmid construction

**Figure 5:**
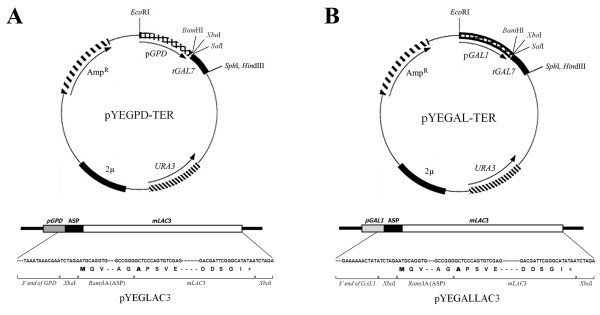
**Schematic diagram of the yeast expression vector for laccase3**. The boxes represent genes or their corresponding functional domains. (**A**) Schematic diagram of the fusion construct cloned into the pYEGPD-TER plasmid and the sequence covering the links of the *GPD *promoter-rice *Amy*1A signal peptides-LAC3. (**B**) Schematic diagram of the fusion construct cloned into the pYEGAL-TER plasmid and the sequence covering the links of the *GAL *promoter-rice *Amy*1A signal peptides-LAC3. The translation start codon and the first codon of LAC3 are in bold. *pGPD*, promoter of glyceraldehyde-3-phosphate dehydrogenase; *pGAL1*, promoter of galactokinase; ASP, rice *amylase1*A signal peptides; *mLAC*3, mature peptide of laccase3; t*GAL7*, terminator of galactose-1-phosphate uridylyltransferase.

A cDNA clone of the *lac*3 gene was obtained from our previous study [11]. The signal peptide (ASP) of amylase 1A (*Ramy1A*) from rice, which has been used in several previous studies to obtain high levels of secretion [33,34], was fused to a mature peptide of the laccase3 protein via overlap extension PCR to create *Xba*I restriction sites (italics) at the 5' and 3' ends using the following primers: forward 5'-GG*TCTAGA*ATGCAGGTGCTGAACACC-3' and reverse 5'-GG*TCTAGA*TTATATGCCC GAATC GTCC-3', and overlap-forward 5'-AACTTGA CAGCCGGGGCTCCCAGTGTCGAG-3' and overlap-reverse 5'-CTCGAC ACTGGGAGCCCCGG CTGTCAAGTT-3' [44,46]. The amplified gene was cloned into a pGEM T-Easy vector (Promega), analyzed via restriction enzyme digestion, and confirmed via DNA sequencing. To construct the yeast expression vector, we excised the ASP/LAC3 fusion fragment from the pGEM T-Easy vector by digestion with *Xba*I and inserted it into a pYEGPD-TER and a pYEGAL-TER vector that harbors the same restriction enzyme site between the glyceraldehyde-3-phosphate dehydrogenase (*GPD*) promoter and the galactose-1-phosphate uridylyltransferase (*GAL7*) terminator and the galactokinase (*GAL1*) promoter and *GAL7 *terminator, respectively [47]. The resultant plasmids were designated as pYEGLAC3 and pYEGALLAC3, respectively (Figure 5).

### Transformation and selection of *S. cerevisiae*

The constructed recombinant vectors were introduced into *S. cerevisiae *2805 according to the lithium acetate procedure [48], and the resulting putative transformants were screened on ura^- ^medium. Colony PCR and back-transformation into *E. coli *using plasmid preparations from putative transformants were performed to confirm the presence of recombinant plasmids in the yeast.

The stability of the introduced plasmids in the yeast was measured as follows. Samples grown in the nonselective YEPD medium were serially diluted with sterile H_2_O to an expected 200 colony-forming units (CFUs) per plate and plated on ura^- ^selective and nonselective plates. Then the relative number of CFUs was determined.

### Laccase assay

The laccase activity in the culture filtrate was determined by spectrophotometric assay using 10 mM 2,6-dimethophenol (DMOP) in sodium tartrate buffer at pH 3.4 as substrate [39]. A laccase unit was defined as a 1.0/min *A*_468 _increase at 25°C as described previously [39].

For the activity staining of native gel, native polyacrylamide gel electrophoresis (PAGE) was performed under non-denaturing conditions [40]; laccase activity was visualized in the gel using DMOP substrate solution.

Tannic acid-supplemented medium was prepared by adding 0.5% (w/v) tannic acid into uracil-deficient selective medium [39]. Then, transformants were grown on the tannic acid-supplemented medium to visualize the laccase activity by coloring the medium.

### Protease inhibition assay

To determine the inhibition of laccase activity due to the presence of protease in cultured media, different types of protease inhibitors, including aprotinin, EDTA, E-64 protease inhibitor, leupeptin, pepstatin, and phenylmethylsulphonyl fluoride (PMSF), were preincubated with cultured media for 10 h at 25°C as recommended previously[41,49]. The preincubated culture medium was then mixed with the culture media containing laccase activity and incubated further for 24 h, after which the residual laccase activity was measured.

### Western blot analysis of expressed laccase3

The partial gene constructs containing the domain from residues 231 to 567 were expressed in *E. coli *as hexahistidine fusion proteins and purified by nickel affinity chromatography according to the manufacturer's directions (Takara). The cDNA encoding the partial laccase3 was amplified by PCR using the primers 5'-GCAG*CTCGAG *CTCGAGGACAACAGC-3' (forward) and 5'-CCC*AAGCTT *TTATATGCCCGA-3' (reverse). The primers were modified to incorporate restriction sites (underlined) for *Xho*I and *Hin*dIII, respectively. The truncated *lac*3 (1014 bp) was inserted into the *Xho*I and *Hin*dIII sites in the expression vectors pColdII DNA (Takara). The resulting recombinant plasmids were transformed into *E. coli *strain BL21. Induction, purification, and confirmation of recombinant laccase3 using anti-hexahistidine antibody were conducted according to the manufacturer's directions (Takara).

Anti-CpLac3 antibody was obtained by injecting 100 μg purified partial laccase3 into a 6-week-old BALB/c mouse, which was boosted with the same amount of the laccase3 emulsified in incomplete Freund's adjuvant 2 weeks after the initial injection. Polysera were obtained 5 days after the booster injection and Western blot analysis was conducted according to standard procedures [45].

## Competing interests

The authors declare that they have no competing interests.

## Authors' contributions

J-MK carried out gene cloning and construction of recombinant yeast strains. S-MP led characterization of recombinant yeast strains. D-HK led the work and wrote the manuscript. All authors read and approved the final manuscript.
